# Antibacterial resistance in ophthalmic infections: a multi-centre analysis across UK care settings

**DOI:** 10.1186/s12879-019-4418-0

**Published:** 2019-09-03

**Authors:** Alice E. Lee, Kanchana Niruttan, Timothy M. Rawson, Luke S. P. Moore

**Affiliations:** 1 Imperial College London, South Kensington Campus, London, SW7 2AZ UK; 2 Chelsea and Westminster NHS Foundation Trust, 369 Fulham Rd, Chelsea, London, SW10 9NH UK; 3 North West London Pathology, Imperial College Healthcare NHS Trust, Fulham Palace Road, London, W6 8RF UK; 4 National Institute for Health Research Health Protection Research Unit (HPRU) in Healthcare Associated Infection and Antimicrobial Resistance, Imperial College London, Commonwealth Building, Du Cane Road, London, W12 0NN UK

**Keywords:** Ophthalmology, Infectious diseases, Microbiology, Conjunctivitis, Antimicrobial resistance, Antimicrobial stewardship, Topical antimicrobials, Eye infections

## Abstract

**Background:**

Bacterial ophthalmic infections are common. Empirical treatment with topical broad-spectrum antibiotics is recommended for severe cases. Antimicrobial resistance (AMR) to agents used for bacterial ophthalmic infections make it increasingly important to consider changing resistance patterns when prescribing, however UK data in this area are lacking. We evaluate the epidemiology and antimicrobial susceptibilities of ophthalmic pathogens across care settings and compare these with local and national antimicrobial prescribing guidelines.

**Methods:**

A retrospective, multi-centre observational analysis was undertaken of ophthalmic microbiology isolates between 2009 and 2015 at a centralised North-West London laboratory (incorporating data from primary care and five London teaching hospitals). Data were analysed using descriptive statistics with respect to patient demographics, pathogen distribution (across age-groups and care setting), seasonality, and susceptibility to topical chloramphenicol, moxifloxacin, and fusidic acid.

**Results:**

Two thousand six hundred eighty-one isolates (*n* = 2168 patients) were identified. The commonest pathogen in adults was *Staphylococcus spp*. across primary, secondary, and tertiary care (51.7%; 43.4%; 33.6% respectively) and in children was *Haemophilus spp*. (34.6%;28.2%;36.6%). AMR was high and increased across care settings for chloramphenicol (11.8%;15.1%;33.8%); moxifloxacin (5.5%;7.6%;25.5%); and fusidic acid (49.6%;53.4%; 58.7%). *Pseudomonas spp*. was the commonest chloramphenicol-resistant pathogen across all care settings, whilst *Haemophilus spp.* was the commonest fusidic acid-resistant pathogen across primary and secondary care*.* More isolates were recorded in spring (31.6%) than any other season, mostly due to a significant rise in *Haemophilus spp*.

**Conclusions:**

We find UK national and local antimicrobial prescribing policies for ophthalmic infections may not be concordant with the organisms and antimicrobial susceptibilities found in clinical samples. We also find variations in microbial incidence related to patient age, clinical setting, and season. Such variations may have further important implications for prescribing practices and modification of antimicrobial guidelines.

## Background

The severity of ophthalmic infections ranges widely from self-limiting bacterial conjunctivitis to potentially sight-threatening conditions such as keratitis and endophthalmitis [1]. One of the commonest ophthalmic infections in the UK is acute infective conjunctivitis, which accounts for approximately 2% of presentations to general practice and constitutes a significant healthcare burden [2].

Antimicrobial resistance (AMR) has been identified as a major healthcare threat worldwide [3] yet few observations have been made regarding the epidemiology and susceptibility patterns of ophthalmic pathogens in the UK [4]. Analysis of electronic data that is routinely collected in healthcare settings is an effective approach for monitoring of AMR [5] and may help to improve clinical management of ophthalmic infections by informing and enabling refinement of current antimicrobial policies.

Whilst antimicrobials are not necessarily required for self-limiting bacterial conjunctivitis, they have been shown to speed symptom resolution compared to placebo, thus reducing the healthcare and societal burden of this common condition [6]. Antimicrobials are also indicated for treatment of corneal abrasion, and bacterial keratitis [7]. Antimicrobial guidelines often recommend empirical antimicrobial treatment in severe infections, often before the results of antimicrobial susceptibility tests are known. However, in some cases empirical prescription of antimicrobials may be with an agent to which the infecting pathogen is resistant, putting the patient at a subsequent risk of treatment failure. This must be balanced against the not infrequent overprescribing of antimicrobials for conditions such as viral or mild bacterial conjunctivitis, for which they are not indicated [8]. Growing international concerns about AMR has led to an increased emphasis on antimicrobial stewardship, a strategy which promotes judicious use of antibiotics to preserve their future efficacy [9]. A key focus of such programmes is to prescribe antimicrobials in relation to local resistance patterns [10], however UK data in this area are lacking.

In this study, we analyse routine microbiological data collected across primary, secondary, and tertiary care settings in London to investigate ophthalmic pathogens and AMR, with reference to local and national antimicrobial policies.

## Methods

### Study setting and design

A retrospective multicentre observational analysis was undertaken to review all clinical microbiology samples sent from eyes/conjunctiva between 2009 and 2015 processed at a centralised North West London microbiology laboratory. This serves a population of 2.5 million people and incorporates data from primary care and five London teaching hospitals including a specialist tertiary ophthalmic hospital. Antimicrobial prescriptions for the pharmacological management of bacterial conjunctivitis are guided by national (for primary care) or local (for secondary and tertiary care) policies (Table 1).

**Table 1:** National and local secondary care antimicrobial prescribing guidelines for the management of bacterial conjunctivitis. Information on national guidelines were adapted from Public Health England [11] guidance. Local guidelines are derived from the Adult and Children Treatment of Eye Infections and Ophthalmology Handbook [7]. Both guidelines highlight the importance of self-care as the initial approach to management of non-severe conjunctivitis, only proceeding to pharmacotherapy where this fails or the infection is severe

Public Health England guidelines for pharmacotherapy for conjunctivitis [11]	Local secondary care guidelines for pharmacotherapy for conjunctivitis [7]
First line: chloramphenicol eye drops (0.5%) or ointment (1%)	First line: chloramphenicol eye drops (0.5%) or ointment (1%)
Second line: fusidic acid (1%) gel	Second line/alternative: moxifloxacin eye drops (0.5%)
	Other options in children:
	• Fusidic acid eye drops (1%) • Erythromycin ointment (0.5%)

Data on patient characteristics, location of patients’ care (primary/secondary/tertiary), specimen type, and organism characteristics (identification, antimicrobial susceptibility pattern) were collected during the period March 2009 to February 2015 from electronic health records and the laboratory management system (LIMS; Sunquest™ Laboratory 7.3.1) containing linked microbiology data. For the tertiary care setting, data were collected during the period March 2012 to February 2015 only. Changes to the laboratory information management system post-2015 precluded data extraction and analysis. All patient data was anonymised. Isolate speciation was performed using API® (bioMérieux) from 2009 to 2011 and matrix assisted laser desorption/ionisation-time of flight (MALDI-TOF) spectroscopy (Biotyper®, Bruker) from 2011 onwards. Antimicrobial susceptibilities were determined by disc diffusion (BSAC criteria) [12]. The minimal level of organism identification was reported in line with the national UK Standards for Microbiology Investigations from Public Health England [13]. As such, pathogens of the family Enterobacteriales were not speciated beyond ‘coliforms’. A minimal number of fungal isolates were identified so the main focus of the study was on bacterial pathogens (excluding mycobacteria).

### Data analysis

The data were de-duplicated (for repeat isolates from the same patient within 14 days) and analysed to describe patient demographics including age and gender. Positive isolates were divided into those obtained from children (< 18 years old) and adults (≥18 years old). The distribution of pathogens was analysed by age-group and level of care (split into primary, secondary, and tertiary care). Pathogen seasonality across each cohort and for each of the most common species was examined. Resistance to chloramphenicol, fusidic acid, and moxifloxacin, three common empirical topical antimicrobials used in bacterial ophthalmic infections, were calculated for each cohort. Resistance was calculated as the proportion of organisms resistant to each agent, including both (i) pathogens identified as intrinsically resistant and (ii) those which were found to be resistant on susceptibility testing. Intrinsically resistant pathogens which also underwent susceptibility testing were removed to avoid data duplication. The following formula was used to calculate resistance rate:

$$ \frac{\mathrm{Intrinsically}\ \mathrm{resistant}\ \mathrm{isolates}+\mathrm{isolates}\ \mathrm{resistant}\ \mathrm{on}\ \mathrm{testing}}{\mathrm{Total}\ \mathrm{isolates}}\ \mathrm{x}\ 100. $$

Descriptive statistics (Chi-square test and Yates correction) were applied where appropriate using SPSS® 24.0 (IBM®, Ca, USA) software.

## Results

### Patient demographics

A total of 2681 ophthalmic isolates from 2168 patients were identified over 6-years. Table 2 details the number of patients and isolates for each care setting as well as patient age and gender distribution. In primary care, 29.5% (433/1467) of isolates were obtained from adults and 70.2% (1030/1467) from children. In secondary care, 39.8% of isolates (297/747) were obtained from adults and 60.2% (450/747) from children. In tertiary care 91.2% isolates (426/467) were obtained from adults and 8.8% (41/467) from children. The number of isolates obtained from adults was significantly higher in secondary and tertiary care than in primary care (*p < 0.01*), whilst the number of isolates obtained from children was significantly higher in primary care than either secondary or tertiary care (*p < 0.01*).

**Table 2:** Demographic details of patients with ophthalmic infections, London, 2009–2015. ^#^age was not reported for four patients

	Care setting		
	Primary	Secondary	Tertiary
Total patients	1209	583	376
Total isolates	1467	747	467
Patient gender, n (%)			
Male	584 (49.3)	339 (58.1)	192 (51.0)
Female	601 (48.7)	244 (41.9)	183 (48.7)
Unknown	24 (2.0)	0 (0.0)	1 (0.3)
Patient age, years			
Mean	20^#^	25	45
Range	0–102	0–99	0–98

### Specimen and pathogen distribution

Eye cultures were obtained from various sources including eye swabs (2358/2681, 88.0%), conjunctival swabs (98/2681, 3.7%), contact lens swabs (54/2681, 2.0%), corneal scrapes (148/2681, 5.5%), and invasive samples (23/2681, 0.8%).

**Fig. 1:**
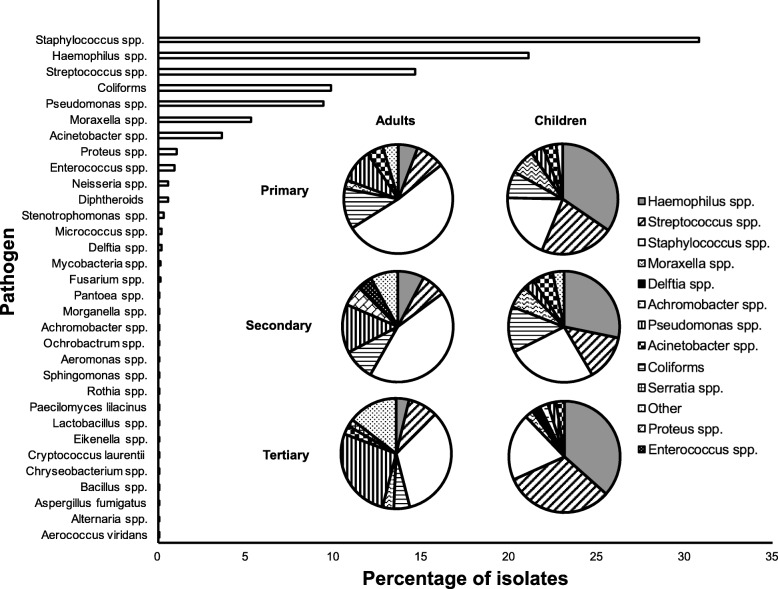
Ophthalmic infection causative organisms, London, 2009–15. Frequency of identification of organisms from clinical ophthalmic microbiology specimens sent to a centralised microbiology laboratory for primary, secondary and tertiary hospitals serving a population of 2.5 million in London. Sub-analysis by age (< 18 years and ≥ 18 years) and level of care (primary, secondary, tertiary) is depicted in the inset pie charts

A wide range of pathogens were identified across all care settings. Figure 1 shows the distribution of pathogens across the cohort and also divided across the primary, secondary, and tertiary care settings.

In adults, the most commonly isolated pathogens were *Staphylococcus spp*. across all levels of care (224/433, 51.7%; 129/297, 43.4%; 143/426, 33.6% respectively). *Staphylococcus aureus* was the most prevalent species of this genus (219/433, 50.6%; 72/297, 24.2%; 86/426, 20.2% respectively), and the contribution of coagulase negative staphylococci to anterior segment ophthalmic infections is unclear. In primary and secondary care, the second most frequent pathogens identified were coliforms (62/433, 14.3%; 48/297, 16.2%, respectively) but in tertiary care was *Pseudomonas spp*. (114/426, 26.8%). The reported prevalence of *Pseudomonas spp.* increased significantly from primary to tertiary care (*p* < 0.01).

In children, in contrast to adults, the most common pathogen across all levels of care was *Haemophilus spp.* (356/1030, 34.6%; 127/450, 28.2%; 15/41, 36.6%, respectively), among which *H.influenzae* predominated (328/356, 92.1%; 118/127, 92.9%; 15/15, 100%). The second most common pathogen isolated in both primary and tertiary care was *Streptococcus spp.* (222/1030, 21.6%; 13/41, 31.7%, respectively), with *Staphylococcus spp.* being the second most frequent pathogen in secondary care (117/450, 26.0%). Compared with adults, *Pseudomonas spp.* isolation in children was significantly less common across all levels of care (in children: 38/1030, 3.7%; 14/450, 3.1%; 1/41, 2.4%, respectively, *p < 0.01* vs. adults). Conversely, *Moraxella spp*. was isolated more commonly from children (76/1030, 7.4%) than adults (11/433, 2.5%) in primary care (*p < 0.01*).

### Seasonality

**Fig. 2:**
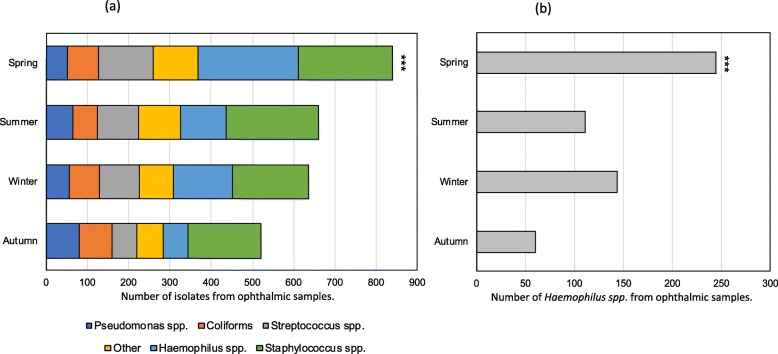
Seasonal variation among ophthalmic infection pathogens, London, 2009–15. 2(**a**) variation across organisms groups, 2(**b**) variation in Haemophilus spp. ***p < 0.001, spring vs. non-spring seasons

Across the cohort, the greatest number of isolates were recorded in spring (848/2681, 31.6%) followed by summer (666/2681, 24.8%), winter (640/2681, 23.9%), and autumn (527/2681, 19.7%)(Fig. 2a**)**. The number of isolates recorded in spring was significantly higher than in other seasons (*p* < 0.001). Across both adult and children in Spring, *Haemophilus spp*. was the commonest organism (245/848, 28.9%; p < 0.001; Fig. 2b), and among children, *Haemophilus spp.* demonstrated an even greater Spring preponderance (197/469; 42.0%).

### Antimicrobial resistance

Rates of antimicrobial resistance to chloramphenicol, moxifloxacin, and fusidic acid increased across care settings (Table 3). The rate of overall chloramphenicol resistance increased significantly from primary to tertiary care (173/1467, 11.8%; 113/747, 15.1%; 158/467, 33.8%; *p* < 0.01). Across all levels of care, the commonest chloramphenicol-resistant pathogen was the intrinsically-resistant *Pseudomonas spp.* (*Pseudomonas spp.* as a percentage of all chloramphenicol-resistant isolates: 82/173, 47.4%; 56/113, 49.6%; 115/158, 75.2%). Among those tested for chloramphenicol susceptibility (i.e. excluding intrinsically-resistant pathogens), those found to be most frequently resistant across all levels of care were the coliforms (as a percentage of non-intrinsically resistant pathogens: 42/91; 46.2%; 36/55; 65.6%; 13/31; 41.9%). Across all levels of care, fewer isolates were tested for moxifloxacin susceptibility than for chloramphenicol or fusidic acid susceptibility. The commonest moxifloxacin-resistant pathogen was again the intrinsically-resistant *Pseudomonas spp*. across all levels of care (*Pseudomonas spp.* as a percentage of all moxifloxacin-resistant isolates: 81/81, 100%primary care; 57/57, 100% secondary care; 115/118, 97.4% tertiary care). For fusidic acid, *Haemophilus spp.* (intrinsically-resistant) was the commonest resistant pathogen in both primary and secondary care, whereas in tertiary care it was *Pseudomonas spp.* Among *Staphylococcus spp.*, rates of fusidic acid resistance were similar between primary (38/429; 8.9%) and secondary care (28/249; 11.2%)(*p* = 0.4) but significantly increased in tertiary care (37/137; 27.0%)(*p* < 0.01).

**Table 3:** Antimicrobial resistance to commonly used topical agents among bacterial isolates from patients with ophthalmic infections, London, 2009–2015. ^#^One isolate was ‘intermediate’. Fusidic acid resistance increased significantly from secondary to tertiary care (p < 0.01). Chloramphenicol resistance increased significantly from primary to tertiary care (*p* < 0.01). ^ including *Pseudomonas spp., Chryseobacterium spp*. ^~^ including Pseudomonas spp. * including enterococci and all Gram-negative pathogens (except *Neisseria spp.* and *Moraxella spp*.) [14, 15]

Antimicrobial	Descriptor	Care setting		
		Primary	Secondary	Tertiary
Chloramphenicol	Intrinsically-resistant isolates^ (n)	5.6% (82)	7.8% (58)	27.2% (127)
	Non-intrinsically resistant isolates undergoing susceptibility testing (n)	94.0% (1301)	91.7% (632)	71.2% (242)
	Non-intrinsically resistant isolates found to be resistant (n)	7.0% (91)	8.7% (55)	12.8% (31)
	Overall antimicrobial resistance	11.8%	15.1%	33.8%
Moxifloxacin	Intrinsically-resistant isolates^~^ (n)	5.5% (81)	7.6% (57)	25.5% (118)
	Non-intrinsically resistant isolates undergoing susceptibility testing (n)	12.5% (173)	7.2% (50)	10.9% (38)
	Non-intrinsically resistant isolates found to be resistant (n)	0% (0)	0% (0)	0% (0^#^)
	Overall percentage resistance	5.5%	7.6%	25.5%
Fusidic acid	Intrinsically-resistant isolates* (n)	46.9% (688)	49.7% (371)	50.3% (235)
	Non-intrinsically resistant isolates undergoing susceptibility testing (n)	55.1% (429)	66.2% (249)	72.0% (167)
	Non-intrinsically resistant isolates found to be resistant (n)	8.9% (38)	11.2% (28)	23.4% (39)
	Overall percentage resistance	49.6%	53.4%	58.7%

## Discussion

The main findings of this study are (i) an age-dependent distribution of ophthalmic infection pathogens with *Staphylococcus spp.* most commonly isolated from adults and *Haemophilus spp.* most commonly isolated from children; (ii) high rates of AMR (intrinsic and acquired) to first line agents for treatment of ophthalmic infections; (iii) difference in infecting pathogens between patients attending primary, secondary and tertiary care, and (iv) a seasonal predilection for ophthalmic infections caused by *Haemophilus spp*. occurring in spring.

Other studies investigating the epidemiology of eye infections in the UK have done so mostly with a narrower focus than the present study, focussing on the microbiology of specific eye infections (i.e. endophthalmitis, keratitis) or rare presentations such as periorbital necrotising fasciitis and phaeohyphomycosis [16–24]. One previous study on adult bacterial conjunctivitis by Silvester et al. evaluated AMR, but focussed on a primary care setting only [4], finding a lower resistance rate to chloramphenicol (8%) than our study (12%) and Silvester et al. concluded chloramphenicol to be a suitable first-line treatment. One possible explanation for this discrepancy points to the lack of coliforms in the study by Silvester et al., which have been previously demonstrated to exhibit reduced susceptibility to chloramphenicol [25]. Conversely, in our study, coliforms represented the greatest proportion of isolates tested and found to be non-susceptible to chloramphenicol. Our study adds to the existing literature by examining AMR across levels of care. Our data reveal increasing AMR to chloramphenicol in the secondary (15%) and tertiary (34%) care settings. This is most likely due to the increasing proportion of intrinsically-resistant *Pseudomonas spp*., which represented up to one quarter of all isolates in the tertiary setting. This may be due to increased contact lens-associated infections seen in tertiary vs. primary care [18, 26]. Another possibility is that patients with *Pseudomonas spp.* infections may have been initially treated empirically in primary care (with non-antipseudomonal agents) and then referred for specialist assessment when the patients did not respond to first-line therapy (a form of ascertainment bias).

In the United States, use of chloramphenicol for bacterial conjunctivitis has been largely superseded by other antimicrobials (including aminoglycosides and fluoroquinolones) due to associations with aplastic anaemia [27]. A large US study investigated in vitro susceptibility to various commonly-prescribed antimicrobials for bacterial conjunctivitis, finding third generation fluoroquinolones (such as moxifloxacin) to be a preferred choice for empirical broad-spectrum coverage, based on resistance rates of both Gram-positive and Gram-negative organisms [28]. In our study, resistance rates to moxifloxacin were approximately half of the respective resistance rates for chloramphenicol in both primary (6% vs. 12%) and secondary (8% vs. 15%) care, but are difficult to interpret because fewer isolates were tested for moxifloxacin susceptibility compared to chloramphenicol or fusidic acid. Less widespread use of empiric topical moxifloxacin in the UK may reflect fears that increasing use of fluoroquinolones will speed up development of resistance, relegating these agents to be ‘last resort’ options [29]. However, previous studies have demonstrated that resistance to moxifloxacin develops slowly as it requires a dual-step mutation [29], suggesting that we should be testing for moxifloxacin susceptibility and possibly using it more often in the UK, as well as monitoring usage trends and resistance.

Another focus of the present study was to analyse the epidemiology of ophthalmic pathogens by patient age. The results show a marked age-dependent distribution of pathogens, with *Staphylococcus spp*. the most common pathogen in adults and *Haemophilus spp*. the most common in children, consistent across all care settings. This could again reflect increased contact lens use in adults [18]. Furthermore, higher prevalence of *Haemophilus spp*. in children may reflect the increased incidence of upper respiratory tract infection and poorer hand hygiene in this age-group.

This variation in causative organisms between age groups has further connotations for empiric antimicrobial prescribing policies. Fusidic acid is commonly used first line in children, yet resistance to fusidic acid was consistently high (50–59%) across all levels of care, driven in part by the preponderance of *Haemophilus spp*. This suggests that prescribers should consider alternative agents in children with better *Haemophilus spp*. cover, particularly in the spring months.

Considering the findings of our study in the context of local and national ophthalmic infection antimicrobial policies, several recommendations may be able to be made. First, given the variation in causative organisms, development of age-dependent antimicrobial guidelines should be strongly considered. We would suggest that particularly among children, where there is a high proportion of *Haemophilus spp*., fuscidic acid is unlikely to be a useful empiric agent. Secondly, the wider use of fusidic acid and chloramphenicol in primary and secondary care should be considered in light of local epidemiology. We would advocate not that these first line therapies necessarily be avoided, but rather that clear early signs of potential antimicrobial failure are highlighted to patients and prescribers, so that early moves to second line agents with lower rates of resistance can be made in a timely fashion. Third, given the variation in AMR, we advocate systems be developed to regularly review ophthalmic infection antimicrobial guidelines in light of local resistance patterns to ensure that first-line therapy is appropriate and effective. Fourth, given resistance rates to all three antimicrobials we examined were unacceptably high (26–59%) in tertiary care (most likely reflecting the high proportion of the intrinsically-resistant *Pseudomonas spp.*), threshold’s for empiric use of anti-pseudomonal agents should be reviewed in specialist eye hospitals. We suggest topical gentamicin or ciprofloxacin may be useful agents, but susceptibility testing for these agents and monitoring of resistance trends must be coupled with any changes to prescribing policies. Finally, whilst prescribing patterns were not examined in this study, the high rates of resistance that we have reported reiterate the importance of restricting antimicrobial use to situations in which they are clinically indicated, in line with national recommendations. This is particularly pertinent given a recent study suggesting that approximately half of UK ophthalmologists may be prescribing antimicrobials for suspected viral conjunctivitis [8].

Our study is limited by its retrospective design and absence of patient level linked clinical data. Whilst samples sent to the microbiology laboratory were for clinical indications (as opposed to being screening or surveillance samples) it is our assumption that these were sent for clinical concerns of infection. We could not ascertain whether the infections (and isolates) were derived from the community or were healthcare associated, although we propose that by far the majority were community associated. Moreover our results will not reflect the sum of all ocular infections seen in primary, secondary or tertiary care as some patients will have been treated empirically and not have had samples sent for culture. This may have biased the results towards more resistant pathogens (either intrinsically or acquired) as it is possible that only those who had already failed first line therapy had samples sent. Therefore we suggest that inferring a need to alter antimicrobial prescribing policies based upon this data must be considered with caution. Whilst routinely collected data, such as that used in our analysis, provides insights into trends in causative organisms and resistance patterns, absolute prevalence can only be obtained by purposeful sampling. To discern the degree with which this limitation may have affected our work, in addition to advocating wider geographical analysis of routinely collected data, we also suggest sentinel surveillance be considered for ophthalmic infections to determine the true burden of causative organisms and resistance. This would further help design of relevant national antimicrobial prescribing guidelines for ophthalmic infections, and would be particularly important for primary and secondary care where there may be a high proportion of patients treated successfully who do not undergo microbiological sampling.

Our study has further limitations in the parsing of data. First, the non-speciation of Enterobacteriales beyond ‘coliforms’ may mask more subtle variations in causative pathogens between age groups and between patients presenting to different care areas. With the advent of low cost rapid speciation platforms, such as MALDI-ToF, we advocate identification of all bacteria from ophthalmic samples to the species level, and have recently adopted this into our operating procedures. Second, we have grouped together anterior segment cultures because of the noted lack of patient level linked clinical data. This precluded us being able to confidently differentiate samples sent for differing clinical diagnoses (conjunctivitis vs. corneal ulcer for example). In the tertiary level care setting having this level of discrimination for syndromic management has obvious benefits when considering construction of empiric antimicrobial policies. However we consider that in primary and secondary care where specialist ophthalmic opinions may not be readily available, empiric antimicrobial prescribing for ophthalmic infections may need to cater for a wider group of presentations. Finally, interpretation of the data was based in vitro susceptibility testing, which may not perfectly predict clinical outcomes. As linked prescribing and clinical outcome data were not available, the relationship between in vitro resistance and treatment failure cannot be determined.

## Conclusion

In our urban UK study analysing the epidemiology of ophthalmic infections we find causative organisms and antimicrobial resistance vary significantly by patient age and care setting. We demonstrate an age-dependent distribution of pathogens and high resistance rates to commonly used antimicrobials, which increases across levels of care. Such findings highlight the need to reassess ophthalmic antimicrobial prescribing policies in the UK in accordance with local resistance patterns. This may include earlier adoption of alternative agents for bacterial conjunctivitis such as moxifloxacin, and tailoring prescribing policies by patient age and clinical setting.

## Data Availability

The datasets analysed during the current study are available from the corresponding author on reasonable request, as long as this meets local ethics and research governance criteria.

## Abbreviations

AMR: Antimicrobial resistance
AMS: Antimicrobial stewardship
BSAC: British society for antimicrobial chemotherapy
LIMS: Laboratory information management system
MALDI-ToF: Matrix assisted laser desorption/ionisation – time of flight
UK: United Kingdom
USA: United States of America
